# Universal partitioning of the hierarchical fold network of 50-residue segments in proteins

**DOI:** 10.1186/1472-6807-9-34

**Published:** 2009-05-20

**Authors:** Jun-ichi Ito, Yuki Sonobe, Kazuyoshi Ikeda, Kentaro Tomii, Junichi Higo

**Affiliations:** 1 Graduate School of Frontier Science, University of Tokyo, 5-1-5 Kashiwanoha, Kashiwa, Chiba, 277-8561, Japan; 2 School of Life Sciences, Tokyo University of Pharmacy and Life Sciences, 1432-1 Horinouchi, Hachioji, Tokyo, 192-0392, Japan; 3 Computational Biology Research Center (CBRC), National Institute of Advanced Industrial Science and Technology (AIST), 2-42 Aomi, Koto-ku, Tokyo 135-0064, Japan; 4 PharmaDesign, Inc., 2-19-8 Hacchobori, Chuo-ku, Tokyo 104-0032, Japan; 5 The Center for Advanced Medical Engineering and Informatics, Osaka University, Open Laboratories for Advanced Bioscience and Biotechnology, 6-2-3, Furuedai, Suita, Osaka 565-0874, Japan

## Abstract

**Background:**

Several studies have demonstrated that protein fold space is structured hierarchically and that power-law statistics are satisfied in relation between the numbers of protein families and protein folds (or superfamilies). We examined the internal structure and statistics in the fold space of 50 amino-acid residue segments taken from various protein folds. We used inter-residue contact patterns to measure the tertiary structural similarity among segments. Using this similarity measure, the segments were classified into a number (*K*_c_) of clusters. We examined various *K*_c _values for the clustering. The special resolution to differentiate the segment tertiary structures increases with increasing *K*_c_. Furthermore, we constructed networks by linking structurally similar clusters.

**Results:**

The network was partitioned persistently into four regions for *K*_c _≥ 1000. This main partitioning is consistent with results of earlier studies, where similar partitioning was reported in classifying protein domain structures. Furthermore, the network was partitioned naturally into several dozens of sub-networks (i.e., communities). Therefore, intra-sub-network clusters were mutually connected with numerous links, although inter-sub-network ones were rarely done with few links. For *K*_c _≥ 1000, the major sub-networks were about 40; the contents of the major sub-networks were conserved. This sub-partitioning is a novel finding, suggesting that the network is structured hierarchically: Segments construct a cluster, clusters form a sub-network, and sub-networks constitute a region. Additionally, the network was characterized by non-power-law statistics, which is also a novel finding.

**Conclusion:**

Main findings are: (1) The universe of 50 residue segments found here was characterized by non-power-law statistics. Therefore, the universe differs from those ever reported for the protein domains. (2) The 50-residue segments were partitioned persistently and universally into some dozens (ca. 40) of major sub-networks, irrespective of the number of clusters. (3) These major sub-networks encompassed 90% of all segments. Consequently, the protein tertiary structure is constructed using the dozens of elements (sub-networks).

## Background

Despite the vast number of amino-acid sequences, protein folds (or superfamilies) are quantitatively limited [1-4]. Consequently, protein fold classification is an important subject for elucidating the construction of protein tertiary structures. A key word to characterize protein folds is "hierarchy". Well-known databases – SCOP [5] and CATH [6] – have classified the tertiary structures of protein domains hierarchically. Similarly, a tree diagram was produced to classify the folds [7].

Mapping the tertiary structures of full-length protein domains to a conformational space, a structure distribution is generated: a so-called protein fold universe [8-11]. A key word to characterize the fold universe is "space partitioning". A two-dimensional (2D) representation of the fold universe was proposed in earlier reports [12,13], where the universe was partitioned into three fold (α, β, and α/β) regions. A three-dimensional (3D) fold universe was partitioned into four fold regions: all-α, all-β, α/β, and α+β [10]. Software that is accessible on a web site, PDBj , serves the distribution on a global surface [14].

The structures of short protein segments have also been studied: Segments of a few (2–3) amino-acid residues long were projected in a two-dimensional (2D) space, where some typical combinations frequently appeared [15]. Fold universes of segments of 4–9 residues long [16] and 10–20 residues long [17-19] showed several clearly distinguishable structural clusters. A systematic survey for 10–50 residue segments has shown that the fold universe is classifiable into segment universes of three types: short (10–22 residues), medium (23–26 residues), and long (27–50 residues) [20]. In this work, the 3D shape of the universe varied abruptly at 23 and 27 residues long. A sequence-structure correlation found in short segments supports the tertiary structure prediction of full-length proteins [21-23].

These studies of protein segments and domains exemplify some structural clusters existing in the low-dimensional (2D or 3D) conformational space. The benefit of the low-dimensional expression is that one can readily imagine the shape of the universe. Increasing the segment length, however, the lowering of the space dimensionality hides the internal architecture of the structure distribution. Consequently, the internal architecture of the distribution for 50-residue segments (or longer segments) is unclear [20]. To compensate the full-dimensional information to the low-dimensional expression, a network is helpful in which two structures close to each other in the full-dimensional conformational space are connected.

Presume an ensemble of points (or nodes). Inter-node linkages form the networks. The network concept has been applied recently to biological systems [24-27]. Structurally similar segments can be linked for the segment fold universe. The structural similarity is computed for the overall structures of two segments (i.e., all coordinates of the segments). Therefore, the similarity is a quantity defined in full-dimensional space. Consequently, a 2D or 3D universe consisting of linked nodes involves full-dimensional information. To assign inter-node linkage in the ensemble, a score is important to quantify the structural similarity between two tertiary structures. Inter-residue contact (native contact) patterns have been used as reaction coordinates in protein folding studies [28-30]. When two structures have similar native contact patterns, they exhibit similar inter-residue packing. Results of several studies indicate that the native contacts are useful indicators to assess the protein folding process [31-43] and folding time scale [41-43].

Herein, we constructed a fold network of 50-residue segments taken from four major structural classes of protein domains. We used the inter-residue contact pattern for the similarity score. The resultant networks showed the main partitioning, as expected. Furthermore, as a new finding, the network of the segment structures was partitioned into dozens of universal communities (sub-networks). From these observations, we propose a novel protein structure hierarchy with community sites at a hierarchy level. The novelty of the currently identified hierarchy was ensured by non-power-law statistics in the hierarchy, which differs from power-law statistics characterizing other hierarchies ever found for protein tertiary structures.

## Results

As described in *Methods*, 50-residue segments were taken from representative proteins and classified into *K*_c _clusters, each of which consists of structurally similar segments. We calculated the native contact patterns that are common in each cluster, and constructed networks by connecting the clusters according to their contact pattern similarity. In *Results*, we first examine the general aspects of the obtained clusters. Second, we check the conformational distribution using a 3D map. Finally, we analyze the characterization of 50-residue segment universe using a network analysis.

As described in this paper, indices *i *and *j *are used for specifying residue positions in a 50-residue segment, *s *and *t *for segment ordinal numbers, *u *and *v *for cluster ordinal numbers, and *w *for a community ordinal number.

### General aspects for clusters

**Figure 1:**
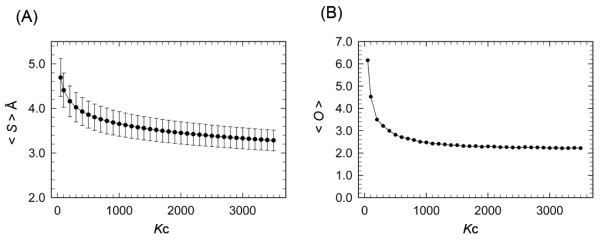
**<*S *> and <*O *> as a function of *K*_c_**. (A) <*S *> is the average cluster size (Eq. 3). The error bar shows the standard deviation over clusters. (B) <*O *> is the average number of segments supplied by a protein to a cluster (see the text for a detailed definition of <*O *>).

Figure 1A portrays the dependence of the average cluster size <*S *> (Eq. 3) on the number *K*_c _of clusters. Actually, *K*_c _determines the spatial resolution to view the universe of the 50-residue segments: With decreasing *K*_c_, <*S *> increases because structurally different segments are fused into a cluster. The change of <*S *> was rapid for small *K*_c _and slow for larger *K*_c_.

The segments were generated by sliding a 50-residue window one residue by one residue along the domain sequences (see *Methods*). Consequently, two segments taken from the same protein domain with mutual adjacency in the sequence might have similar structures and might therefore be involved in a cluster. We did the following analysis to verify this possibility quantitatively: Presume that a cluster *u *involves *n*_*m *_segments originated in a protein *m*. Subsequently, we introduced a quantity: , where the summation is taken over proteins that supply segment(s) to the cluster *u*, and *N*_p _is the number of those proteins. Figure 1B presents a plot of the average of *O*_*u *_as a function of *K*_c_: . For *K*_c _= 1000, <*O *> converged to 2.2. Consequently, a protein supplies only two or three segments to a cluster on average: i.e., a cluster does not contain excessive segments derived from a single protein for *K*_c _≥ 1000.

**Figure 2:**
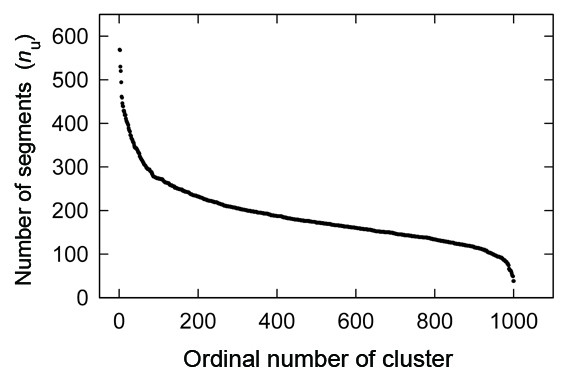
**Number *n*_*u *_of segments in a cluster as a function of the ordinal number of the cluster**.

Figure 2 depicts the number (*n*_u_) of segments involved in a cluster as a function of the cluster ordinal number for *K*_c _= 1000. The decay of *n*_u _is non-exponential. It is particularly interesting that even cluster #950 involves more than 100 segments, which means that the cluster comprises more than 40 (= 100/2.5) different proteins (<*O *> ≈ 2.5 for *K*_*c *_= 1000). In the last 50 clusters, *n*_u _decreased quickly. These clusters consist of randomly structured segments. Although segments were taken from all-α, all-β, α/β, and α+β SCOP classes, the structures can be random.

**Figure 3:**
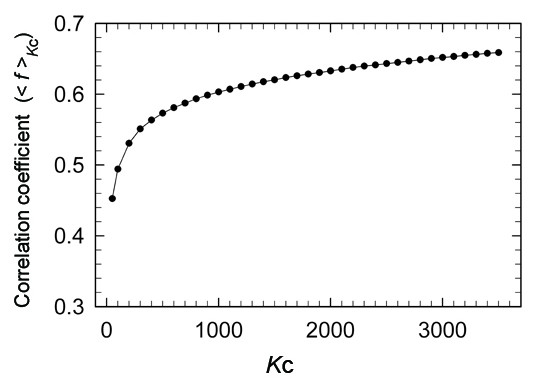
**Averaged correlation coefficient <*f *>_*K*c _(Eq. 9) for intra-cluster segments as a function of *K*_c_**.

Figure 3 depicts <*f *>_*K*c _(Eq. 9) depending on *K*_c_. The value of <*f *>_*K*c _was 0.60–0.65 for *K*_c _≥ 1000. The similarity threshold *f*_0 _for assigning the inter-cluster linkage (Eq. 7) was 0.7. Figure 3 presents that the inter-residue similarity is compatible with the intra-cluster similarity.

### Fold universe and network of clusters

The inter-cluster (inter-node) links were assigned to the *K*_*c *_clusters according to the adjacency matrix *a*_*uv*_. Directly connected clusters have mutually similar inter-residue contact patterns. Internal architectures of the networks were investigated by dividing the networks into communities (sub-networks) using Newman's method [44]. In parallel, we projected the networks into a 3D space to obtain positions in the conformational space (see Additional file 1 for details). Although the clusters were embedded in the 3D space, the inter-cluster links were given to clusters that are mutually close in the full-dimensional space.

Each community was characterized by five biophysical structural features: the α, β, αβ secondary-structure elements, the radius of gyration, and the number of inter-residue contacts, denoted respectively as *n*_*α*_, *n*_*β*_, *n*_*αβ*_, *R*_g_, and *N*_contact_. Then, the communities were classified into four types (α, β, αβ, and randomly structured communities) depending on the five structural features (see *Methods *for details).

**Figure 4:**
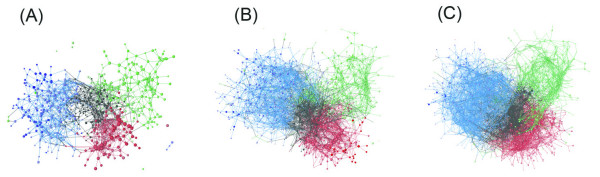
**Networked 3D distribution of clusters for *K*_c _= 1000 (A), 2000 (B), and 3000 (C)**. In this figure, a sphere represents a cluster. The larger the sphere, the more segments the cluster involves. The coloring method for clusters and inter-cluster links is explained briefly below (see Additional file 1 for details): The α, β, and αβ communities are, respectively, red, blue, and green. The larger the secondary-structure contents in a community, the greater the color strength. All randomly structured communities are shown in black. Colors assigned to cluster-cluster links are as follows: red for links within α communities, blue for those within β communities, green for those within αβ communities, and black for other links.

**Figure 5:**
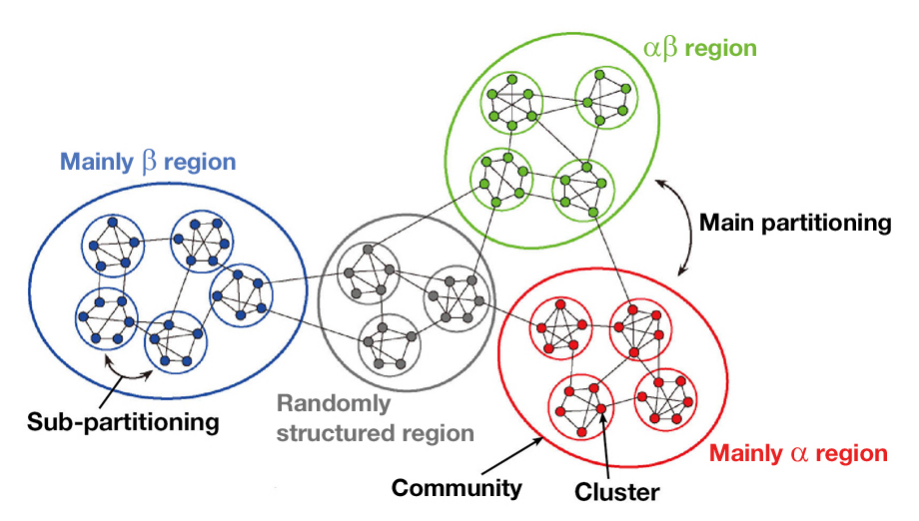
**Main and sub-partitioning of the cluster network**.

Figure 4 portrays the 3D cluster distributions at *K*_c _= 1000, 2000, and 3000, where a single color was assigned to a community depending on secondary-structure elements *n*_*α*_, *n*_*β*_, and *n*_*αβ *_(see Additional file 1 for details). This figure clearly illustrates that the 3D cluster network is partitioned into four fold-regions (mainly α, mainly β, αβ, and randomly structured regions) independent of *K*_c_, which respectively consist of α, β, αβ, and randomly structured communities. We termed this partitioning as "main partitioning". Figure 5 shows that the overall shape of the network adopted a three-leaf clover shape (mainly α, mainly β, and αβ regions surrounding the randomly structured region). We checked quantitatively whether the 3D distribution reflected the original full-dimensional distribution by calculating F-measure  (see Additional file 1 for the definition of ). The value of  was, respectively, 0.804 for *K*_c _= 1000, 0.673 for *K*_c _= 2000, and 0.593 for *K*_c _= 3000. The large value of  for *K*_c _= 1000 indicates that the 3D cluster distribution fairly reflects the full-dimensional distribution. The  value decreased concomitantly with increasing *K*_c_. However, the three-leaf clover shape of the distribution was conserved at various *K*_c_, which strongly suggests that the main partitioning exists in the 50-residue segments universe.

**Figure 6:**
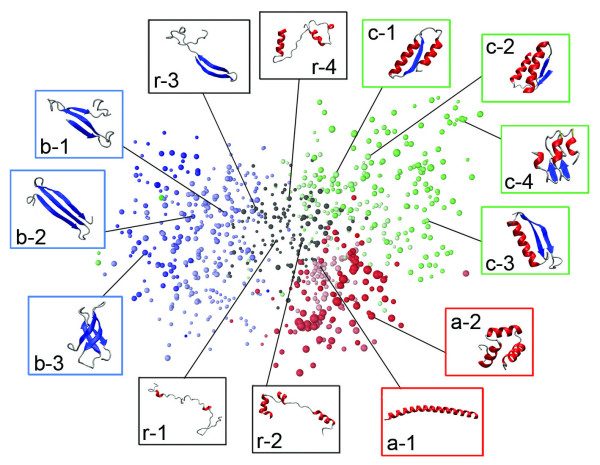
**Tertiary structures picked from 3D distribution for *K*_c _= 1000 Colors**. of clusters are the same as those depicted in Figure 4. Inter-cluster links are not shown. This figure is presented with the same orientation as that of Figure 4.

**Figure 7:**
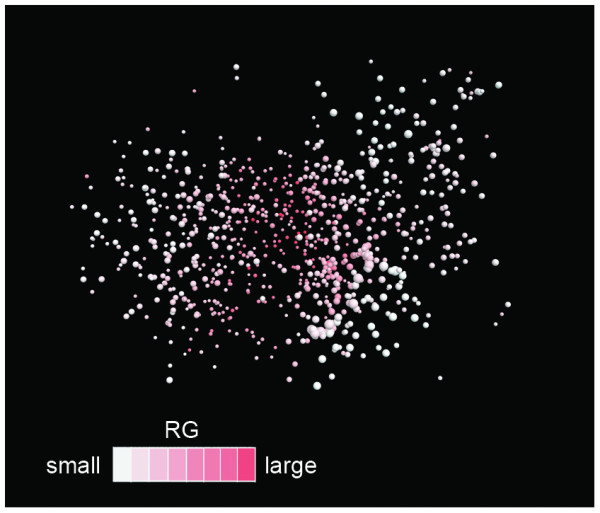
**Radius of gyration *R*_g _of clusters**. With increasing *R*_g_, the cluster color is redder. This figure is presented with the same orientation as that of Figure 4.

Figure 6 displays segment tertiary structures picked from clusters. This figure portrays that the structure classification by the five structural features correlates well with the visual secondary-structure constitution. Most segments originating in the all-α SCOP fold class were assigned to the α communities (see a-1 and a-2 in Figure 6). Those that originated in the all-β SCOP fold class were assigned to the β communities (see b-1 – b-3). The majority of segments taken from the α/β SCOP fold class were assigned to the αβ communities (see c-1 – c-4), although some were involved in other fold regions. In contrast, segments from the α+β SCOP fold class scattered to all the fold regions because the α+β proteins are a mixture of helices, strands, and randomly structured fragments, where the α and β secondary-structure elements are not necessarily neighbors to each other in the sequence. Consequently, the 50-residue segments from the α+β proteins can involve various structural features. The randomly structured region contained clusters with a few secondary-structure elements (see r-1 – r-4 in Figure 6). However, its polypeptide packing was loose, as portrayed in Figure 7, where the randomly structured clusters had large *R*_g_.

### Non-power-law statistics

The protein-domain universe is known to be an extremely biased distribution [8,45]. Many studies have suggested a power-law statistic to represent the relation between the number of families and the number of folds [9,46,47]. For instance, Shakhnovich and co-workers created a protein-domain universe graph (PDUG) with adoption of a DALI Z-score for the similarity score, and showed that the domain universe followed a power-law distribution [9]. Consequently, it is interesting to check if the currently produced network of the 50-residue segments follows the power law distribution.

**Figure 8:**
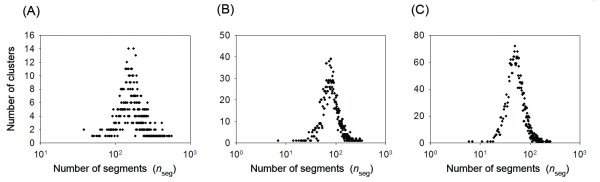
**Relation between number (*n*_seg_) of segments involved in a cluster and number of clusters for *K*_c _= 1000 (A), 2000 (B), and 3000 (C)**.

First, we calculated the number (*n*_seg_) of segments involved in each cluster. Figures 8A, B, and 8C portray the relation between *n*_seg _and the number of clusters that respectively involve *n*_seg _segments at *K*_c _= 1000, 2000, and 3000. The distributions were symmetric (the value of skewness was 0.138 for *K*_*c *_= 1000, 0.006 for *K*_*c *_= 2000, and -0.066 for *K*_*c *_= 3000) on the X-axis, log(*n*_seg_), and far from the power-law statistics. Therefore, the currently obtained universe differs from those that have ever been reported. Additionally, we calculated the number (*n*'_*seg*_) of segments involved in each community, and showed the relation between *n*'_*seg *_and the number of communities involved *n*'_*seg *_fragments for *K*_c _= 1000, 2000, and 3000. We again obtained non-power-law statistics in the relation (data not shown).

**Figure 9:**
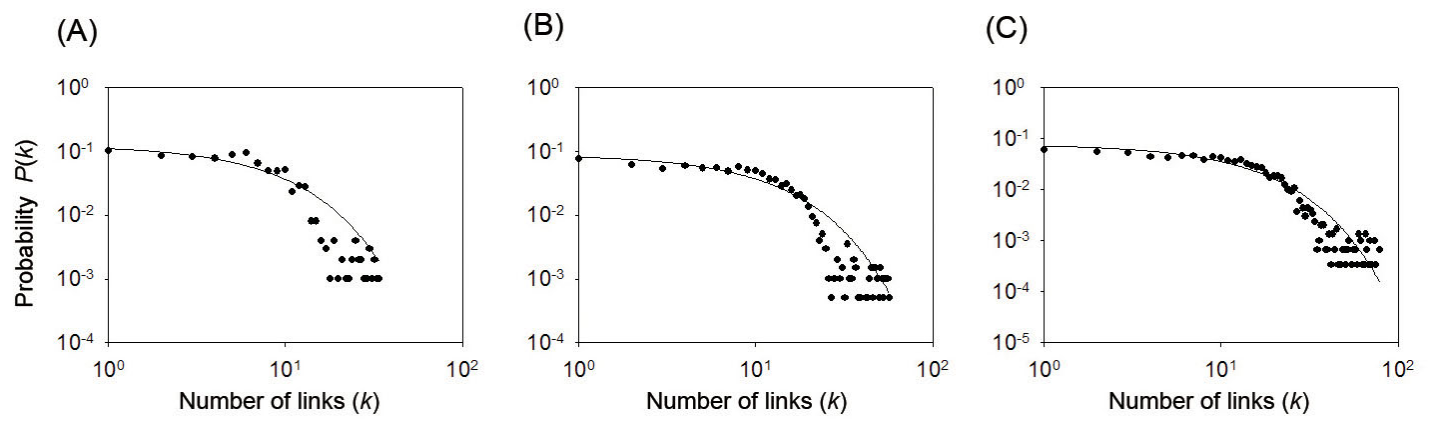
**Connectivity distribution *P*(*k*) of cluster network at *K*_c _= 1000 (A), 2000 (B), and 3000 (C)**. The X-axis *k *shows the number of links of a cluster connected to other clusters. Solid lines are the best-fit curves drawn assuming that *P*(*k*) decays with *k *exponentially.

Next, we calculated a connectivity distribution, *P*(*k*), of the networks to investigate details of the cluster network [48]. The *P*(*k*) is defined as a distribution function of clusters that have *k *links to other clusters. Figures 9A, B, and 9C respectively present *P*(*k*) at *K*_*c *_= 1000, 2000, and 3000. Subsequently, *P*(*k*) decays exponentially with increasing *k*. Therefore, these distributions are exponential ones (or possibly truncated power-law distributions). Consequently, non-power-law networks (i.e., non-scale-free networks) are again observed for the current networks.

### Robustness of communities

**Figure 10:**
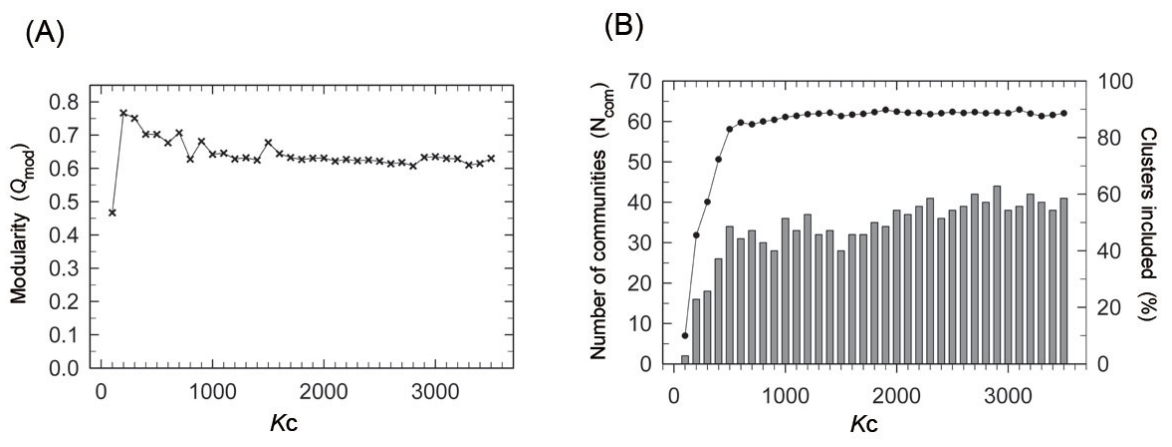
***K*_c _dependence of *N*_com _and *Q*_mod_**. (A) The *K*_c _dependence of modularity *Q*_mod _(Eq. 10). (B) The bar graph shows the *K*_c _dependence of number, *N*_com_, of communities assigned to the left y-axis. The line with filled circles represents the ratio (assigned to right y-axis) of clusters in major communities to all clusters.

We conducted modularity analysis to study cluster networks from another perspective. First, the networks were divided into communities (see *Methods*). A modularity *Q*_mod _is an index to assess how well the network is divided into communities [49]: 0 ≤ *Q*_mod _≤ 1. A network with a large *Q*_mod _is characterized by numerous intra-community links and a few inter-community links. Figure 10A portrays the *K*_c _dependence of *Q*_mod_, which has the maximum at *K*_c _= 200, indicating that the communities were highly isolated at *K*_c _= 200. For *K*_c _> 200, the communities were connected gradually by links, thereby decreasing *Q*_mod_. For *K*_c _≥ 1000, *Q*_mod _converged to a value (0.63), which indicates that the 50-residue segment network is characterized by high modularity.

We next calculated the number of communities at various *K*_c_. We classified the communities into major and minor communities. Major ones are communities consisting of more than three clusters. Then, minor ones are small isolated communities consisting of only one or two clusters without links to other communities. No community involves only one cluster linked to another community. The *K*_c _dependence of the number (*N*_com_) of the major communities is presented in Figure 10B. The minor communities do not characterize the overall property of the network because only 10% of clusters belong to the minor communities at any *K*_c_. The increment of *N*_com _with increasing *K*_c _was rapid for 100 ≤ *K*_c _≤ 1000 and slow for *K*_c _≥ 1000. The values of *N*_com _were, respectively, 36, 38, and 38 at *K*_c _= 1000, 2000, and 3000. This result shows that the number of communities was conserved for *K*_c _≥ 1000.

**Figure 11:**
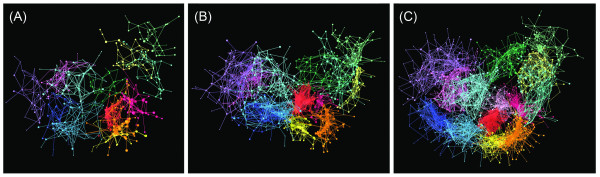
**Communities at *K*_c _= 1000 (A), 2000 (B), and 3000 (C)**. For each universe, only the top 13 communities by the number of involved clusters are shown. A single color is assigned to communities that are common to the three universes. Communities that are not common among the three are not shown, nor are minor communities.

In addition to the analysis presented above, we checked to determine whether the contents (i.e., segments) involved in the communities are conserved with variation of *K*_c_. Subsequently, we assigned a single color to communities common to the universes at *K*_c _= 1000 (Figure 11A), 2000 (Figure 11B), and 3000 (Figure 11C). For instance, the majority of segments in the orange community of Figure 11A were involved in the orange ones in Figures 11B and 11C. Consequently, the communities are conserved well in the universes at different *K*_c_. In other words, the network partitioning into communities is universal, independent of the spatial resolution (i.e., *K*_c_). We termed this inter-community partitioning as "sub-partitioning", whereas the main partitioning is inter-regional partitioning (Figure 5).

## Discussion

**Figure 12:**
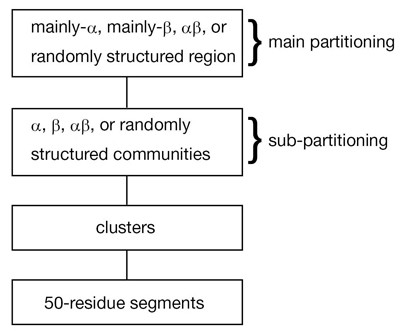
**Hierarchy in the segment universe proposed from the current study**.

Herein, we described universal partitioning of two types in the 50-residue segment networks (Figure 5) based on the network analysis. The main partitioning (the network separation by fold regions) resembles that in the classification scheme of existing databases such as CATH and SCOP. The mainly α, mainly β, αβ, and randomly structured regions consist respectively of α, β, αβ, and randomly structured communities. However, for the first time, we found communities in the segment fold universe: this sub-partitioning (network separation by communities) is a novel finding. High modularity ensures persistently existing communities, where the intra-community clusters are linked tightly and the inter-community clusters are linked weakly. The universality of the sub-partitioning was remarkable for *f*_0 _(0.65 ≤ *f*_0 _≤ 0.75). Nevertheless, outside this range, the universality vanishes gradually. Our results reveal a hierarchically structured universe for 50-residue segments, as depicted in Figure 12. This hierarchy is robust because the main and sub-partitionings are independent of *K*_c _for *K*_c _≥ 1000.

Figure 10B portrays that the current universe for the 50-residue segments consists of some dozens (ca. 40) of major communities. Kihara and Skolnick reported that the current PDB database might cover almost all structures of small proteins [50]. Crippen and Maiorov generated many self-avoiding conformations of a chain and suggested that the possible structures of a 50-residue chain are classifiable roughly into a small number of types, although the secondary-structure formation was not incorporated in their model [51]. A study proposed the conjecture that tertiary-structure evolution of proteins might be achieved using limited repertoires of basic units such as supersecondary structure elements [52]. Results of such studies are consistent with our results because we have shown that protein tertiary structures can be decomposed into the dozens of major communities of 50-residue segments. Actually, 90% of clusters belong to the major communities. To link those studies with our study more closely, detailed contents of each major community should be investigated. In fact, such a research project is proceeding now. Moreover, the role of the minor communities in the protein structure construction should be studied.

The currently observed 50-residue segment universe was characterized by the non-power-law distribution. Our result apparently differs from the power-law distribution widely known for the hierarchical protein domain universe [9,46,47,53]. The emergence of the non-power-law statistics might be related to the usage of the inter-residue contact, which is a more relaxed similarity measure than widely used ones such as RMSD or the DALI Z-score. It is known that in the power-law statistics the rate for isolated clusters in the entire clusters is high [53]. In our non-power law statistics, the rate was low because the relaxed measure provided linkages between clusters. Thus, the two statistics compensate to each other to survey the fold universe. From the non-power-law universe, we could show a novel hierarchy (Figure 12) in the universe and the existence of 40 repertories (Figure 10) to construct the protein tertiary structures, which have not been reported from the power-law universe. These results were also found in the 60- and 70-residue segment universes (data not shown). This suggests that the non-power law is likely to be a general property for segment universes.

The current network helps to trace conformational changes of segments along the network linkages. *Supplementary Results *displays that the conformation gradually changes when shifting the view from a cluster to another (see Additional file 1).

The inter-residue contact (native contact) has been widely used as a reaction coordinate in protein folding (see *Introduction*). We intend to use the currently obtained networks for protein folding study. The networks of fixed-length segments are readily applicable for conformational sampling in protein folding, where the chain length is usually fixed. The randomly structured clusters are located at the root of the distribution (Figure 4 and Figure 5), from which the segment conformation can diversify to mainly α, mainly β, or αβ regions with increased compactness (Figure 7).

## Conclusion

We constructed a 50-residue segment network for investigating the protein local structure universe. The network was partitioned into some dozens (ca. 40) of major communities with high modularity (0.60 <*Q*_mod _< 0.65), independent of the spatial resolution (*K*_c_). The major communities existed universally and persistently in the universe. Surprisingly, 90% of all segments were covered by the major communities. Consequently, numerous similarities exist among local regions (i.e., 50-residue segments) of proteins. Furthermore, the currently constructed segments networks are characterized by non-power-law (non-scale-free) statistics, which apparently differs from reported characteristics for the fold universe of full-length proteins.

## Methods

This section includes six subsections. The first three – "Generation of 50-residue segment library", "Clustering segments", and "Computation of inter-residue contact patterns" – are preparative subsections describing construction of the 50-residue segment fold universe. In the subsection titled "Construction of a universe and network", construction of the fold universe and the network is described. "Modularity analysis" presents analyses used to examine the network. The subsection "Characterization of communities by structural features" describes a method to characterize communities depending on five structural features. Specification of indices *i*, *j*, *s*, *t*, *u*, *v*, and *w *is given at the beginning of *Results*.

### Generation of 50-residue segment library

We generated a structure library of 50-residue segments with reference to the all-α, all-β, α/β, and α+β fold classes defined in the SCOP database (release 1.69) [5]. The SCOP database presents a list that provides a representative for each protein family. We selected tertiary structures of the representative domains from the PDB database [54] with elimination of multi-chain domains, those involving structurally undetermined regions, and those shorter than 50 residues. Furthermore, we eliminated domains consisting of 400 residues or more, which might involve structurally repeating units. Then we obtained 1803 domains (456 from all-α, 393 from all-β, 393 from α/β, and 561 from α+β). A domain that is *n*_r _amino-acid residues long produces *n*_r _- 49 segments from sliding a 50-residue window along the sequence one residue-by-one residue. Finally, we obtained an ensemble of 186 821 segments (32 040 from all-α, 39 375 from all-β, 63 177 from α/β, and 52 229 from α+β). The residue site of each segment was re-numbered from 1 to 50 in our study.

### Clustering segments

We classify the collected segments into clusters as follows: First, the inter-C_α _atomic distances were calculated for segment *s*, where the distance between residues *i *and *j *is denoted as *r*_*s*_(*i*, *j*). We eliminated residue pairs |*i *- *j*| < 3 because the distances for these pairs are similar for all segments. In other words, those distances have less sensitivity to discriminate the structural differences of segments. Then, the number (*N*_pair_) of the C_α_-atomic pairs in a 50-residue segment is 1128: *N*_pair _= 1128. The set of distances is expressed as a *N*_pair_-dimensional vector:  = [*r*_*s*_(1, 4), *r*_*s*_(1, 5), ..., *r*_*s*_(47, 50)]. We define the root mean square distance (*rmsd*_*st*_) between  and  as in the *N*_pair_-dimensional Cartesian space: .

For classifying the 186 821 segments into *K*_c _clusters, we applied Lloyd's K-means algorithm [55] to the set of *rmsd*_*st *_values, where *s*, *t *= 1, ..., 186821. One should set *K*_c _in advance in the K-means algorithm. We examined various values for *K*_c _(*K*_c _≤ 5000). In Lloyd's method, the *K*_c _clusters are set randomly at the beginning. The finally converged clusters are output. We have checked that the main results are independent of the initial set of clusters.

We calculated the center () of a cluster *u *in the *N*_pair_-dimensional space as , where the element  is given as

(1)

The *n*_*u *_is the number of constituent segments of the cluster *u*.

We defined a size *S*_*u *_of the cluster *u *as

(2)

This equation simply quantifies the average distance from the cluster center  to segments belonging to the cluster *u *in the *N*_pair_-dimensional space. The average cluster size is defined simply as

(3)

where the summation is taken over all the *K*_c _clusters.

### Computation of inter-residue contact patterns

In this subsection, we present computation of the inter-cluster and intra-cluster structural similarity based on the inter-residue contact patterns. The inter-residue contacts in segment *s *were defined as follows: Calculating all the inter-heavy atomic distances between residues *i *and *j *for the segment, their minimum distance was registered as the inter-residue distance *q*_*s*_(*i*, *j*). Then, if *q*_*s*_(*i*, *j*) < 6.0 Å, we judged that the residues *i *and *j *were contacting and set a quantity *c*_*s*_(*i*, *j*) to 1 (otherwise, *c*_*s*_(*i*, *j*) = 0). Here, we again eliminated residue pairs of |*i *- *j*| < 3 in the calculation of *c*_*s*_(*i*, *j*). The set of *c*_*s*_(*i*, *j*) constructs a matrix *C*_*s*_, where element (*i*, *j*) is *c*_*s*_(*i*, *j*).

The upper limit (6.0 Å) for *q*_*s*_(*i*, *j*) allows no penetration of a water molecule between residues *i *and *j*: At *q*_*s*_(*i*, *j*) = 6.0 Å, the substantial space for water penetration between the residues is approximately 2.0 Å (= 6.0 - 2 × 2.0) assuming that radii of segment heavy atoms are 2.0 Å. This space of 2.0 Å is smaller than the diameter of a water molecule (2.8 Å).

A structural similarity between segments *s *and *t *might be counted by comparing *C*_*s *_and *C*_*t*_. However, a strict comparison engenders an oversight of the similarity in the following case: Presume that *c*_*s*_(*i*, *j*) = 1 and *c*_*t*_(*i*,+ 1, *j*) = 0 in the segment *s*, and *c*_*s*_(*i*, *j*) = 0 and *c*_*t*_(*i*,+ 1, *j*) = 1 in segment *t*. The inter-residue contacts in these segments differ but they are similar. The strict comparison does not count such a similarity. To incorporate such similarity, smoothing of *C*_*s *_was performed as

(4)

**Figure 13:**
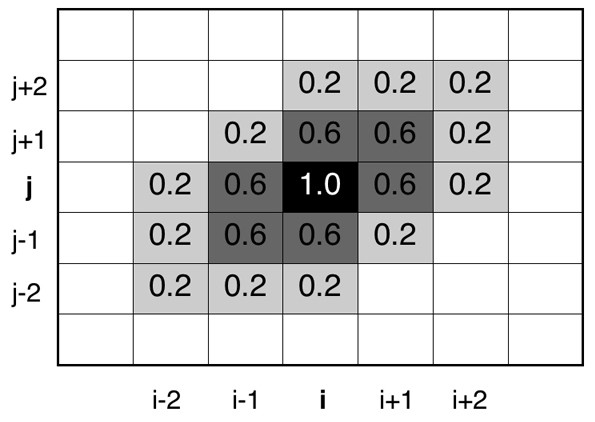
**Smoothed inter-residue contacts *c*(*i*, *j*) (Eq. 4)**. It is presumed that residue pair (*i*, *j*) is in contact (i.e., *c*(*i*, *j*) = 1), and that the other pairs are non-contacting. Equation 4 provides negative *c*_*s*_(*i'*, *j'*) at sites where an inequality, |*i *- *i'*| + |*j *- *j'*| + |(|*i *- *i'*| - |*j *- *j'*|)| > 5, is satisfied. Besides, this inequality is satisfied without exception when any one of the three inequalities, |*i *- *i'*| > 2, |*j *- *j'*| > 2, or ||*i *- *i'*| - |*j *- *j'*|| > 2, is met. Those negative *c*(*i*, *j*) = 1), and that the other pairs are non-contacting. Equation 4 provides negative *c*_*s*_(*i'*, *j'*) are reset to zero (see text).

This smoothing (see Figure 13) was done only when residues *i' *and *j' *are not contacting and the residues *i *and *j *are contacting in the segment. If Eq. 4 produces a negative value, then *c*_*s*_(*i'*, *j'*) is set to zero. If a non-contacting residue pair (*i'*, *j'*) has multiple values for *c*_*s*_(*i'*, *j'*) attributable to contributions of some contacting pairs around (*i'*, *j'*), then the largest value is assigned to the non-contacting pair. As described in this paper, the inter-residue contact matrix *C*_*s *_indicates that after the smoothing.

Here, we calculate the contact patterns which are specific to a cluster. For this purpose, we averaged *C *over the entire segment library and over all segments in cluster *u*. We denote these averaged matrices as  and , respectively. Then, we defined a quantity , where element (*i*, *j*) is denoted as . The similarity between clusters *u *and *v *was measured using the following correlation coefficient:

(5)

where

(6)

The term  in Eq. 5 is defined by setting *u *= *v *in Eq. 6, and the term  by setting  = 1. A large correlation coefficient indicates similar inter-residue contact patterns between the clusters.

The coefficient  is useful as a distance between clusters *u *and *v *in a multi dimensional space. Consequently, the set of coefficients define a multi-dimensional weighted graph (i.e., weighted network). In this work, we must convert this weighted graph into an un-weighted one to perform community analysis, which only deals with the un-weighted graph. Therefore, we introduce an adjacency matrix *a*_*uv *_in which element (*u*, *v*) is given as follows.

(7)

The inter-residue contact patterns are similar between clusters *u *and *v *only when . Herein, we set *f*_0 _to 0.7. The meaning of 0.7 is explained in the *Results *section.

We next assessed the intra-cluster similarity. First, we defined a quantity  for a segment *s*, where element (*i*, *j*) of Δ*C*_*s *_is denoted as Δ*C*_*s*_(*i*, *j*). Then, we averaged Δ*C*_*s*_(*i*, *j*) over the segments in cluster *u*:

(8)

We define a matrix *G*_*u *_for that the element (*i*, *j*) as *g*_*u*_(*i*, *j*). Then, we calculated the correlation coefficient *f*(*G*_*u*_, Δ*C*_*s*_) between *G*_*u *_and Δ*C*_*s *_for segments in cluster *u*, using the same definition as that in Eq. 5. Subsequently, we calculated an averaged correlation coefficient <*f *>_*u *_over *f*(*G*_*u*_,Δ*C*_*s*_) of the segments in the cluster *u*. This quantity is a measure to express the similarity of the inter-residue contact patterns among the segments in cluster *u*. Finally, <*f *>_*u *_was averaged over all clusters.

(9)

The larger the value of , the more similar the inter-residue contact patterns in each cluster are, on average.

### Construction of a universe and network

We constructed a distribution (i.e., fold universe) of *K*_c _clusters in a 3D conformational space with embedding clusters into the 3D. Details are presented in Additional file 1. As explained in the *Introduction*, lowering of the space dimensionality hides the internal architecture of the fold universe. To compensate the full-dimensional information to the 3D distribution, links were assigned to clusters with similar inter-residue contact patterns (*a*_*uv *_= 1). The generated networks were subjected to the modularity analysis described in the next subsection.

### Modularity analysis

**Figure 14:**
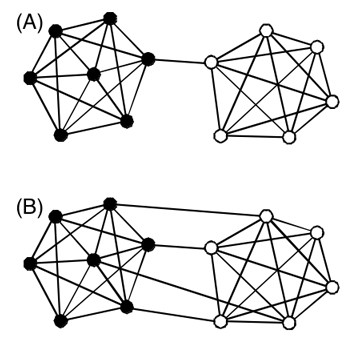
**Two network types**. Network (A) has larger modularity *Q*_mod _than (B) does. Filled circles form a community (Com 1); open ones construct the other community (Com 2). Lines between circles represent links.

To investigate a property of the cluster network, we divided the network into communities (i.e., sub-networks) using an efficient method [44]. An example of a network is presented in Figure 14, where two communities (Com 1 and Com 2) exist. A modularity *Q*_mod _is an index to assess how well the network is divided into communities [49]:

(10)

where *I*_*w *_is the number of links connecting clusters within a community *w*, *N*_com _is the number of communities existing in the entire network, and *I *is the number of links existing in the entire network. The quantity *d*_*w *_is called the "total degree", which is defined for each community as *d*_*w *_= 2*I*_w _+ *I*_w-other_, where *I*_w-other _is the number of links connecting clusters in the community *w *and clusters outside the community. The value of *Q*_mod _is 0–1: *Q*_mod _approaches 1 when the number of links connecting different communities decreases. For instance, the network in Figure 14A has *Q*_mod _of 0.466 (*I *= 34, *I*_1 _= 18, *I*_2 _= 15, *d*_1 _= 37, and *d*_2 _= 31). That of Figure 14B has *Q*_mod _of 0.388 (*I *= 37, *I*_1 _= 18, *I*_2 _= 15, *d*_1 _= 40, and *d*_2 _= 34). The two networks are equivalent except for the inter-community links.

### Characterization of communities by structural features

The manner of differentiating the communities is important. Herein, we characterize the communities depending on five biophysical structural features: radius of gyration (*R*_g_), number of inter-residue contacts ( with removal of pairs of |*i *- *j*| < 3), number of α-helical residues (*n*_*α*_), number of β-helical residues (*n*_*β*_), and the sum of *n*_*α *_and *n*_*β *_(i.e., *n*_*αβ *_= *n*_*α *_+ *n*_*β*_).

First, we calculate the five quantities for each segment. The secondary-structure assignment to each residue in a segment is done using software available at the STRIDE web site [56]. Next, we took the average for each of the five quantities over segments in a community. We designate the average quantities in a community *w *as *R*_g_(*w*), *N*_contact_(*w*), *n*_*α*_(*w*), *n*_*β*_(*w*), and *n*_*αβ*_(*w*). Then, we classify the communities into α, β, αβ, and randomly structured ones according to the five quantities: Randomly structured communities are those with *R*_g _> 14 Å and *N*_contact_(*w*) < 100 or those with *n*_*αβ*_(*w*) < 15. In the remaining communities, α communities are those with *n*_*α*_(*w*) > 0.7 × *n*_*αβ*_(*w*). In the remaining communities, β communities are those with *n*_*α*_(*w*) > 0.7 × *n*_*αβ*_(*w*). The finally remaining communities are classified as αβ communities. Each segment in the αβ communities significantly involves both an α helix and a β strand.

## Authors' contributions

This study was conceived and carried out by JI, who also developed the main part of the methodology. YS participated in some analyses. IK participated in discussions. KT participated in the coordination of the study. He also helped to write the manuscript. JH participated in developing the methodology, designed the study, and wrote the manuscript. All authors read and approved the final manuscript.

## Supplementary Material

Additional file 1 **Supplementary Methods and Supplementary Results**. There are three sections in the Supplementary Methods as follows: (1) The method of embedding the inter-cluster network into 3D space. (2) The definition of F-measure. (3) The coloring method for clusters in the 3D network. In the Supplementary Results, tertiary structures of fragments in the same cluster and those in the same community are discussed. Click here for file
