# Supplementary material for: Universal partitioning of the hierarchical fold network of 50-residue segments in proteins
Source: BMC Struct Biol. 2009 May 20;9:34. doi: 10.1186/1472-6807-9-34 (PMC2693521; doi:10.1186/1472-6807-9-34)
Supplement: Additional file 1 — Supplementary Methods and Supplementary Results. There are three sections in the Supplementary Methods as follows: (1) The method of embedding the inter-cluster network into 3D space. (2) The definition of F-measure. (3) The coloring method for clusters in the 3D network. In the Supplementary Results, tertiary structures of fragments in the same cluster and those in the same community are discussed. [file 1472-6807-9-34-S1.doc]

**Universal partitioning of the hierarchical fold network of 50-residue segments in proteins**

### Jun-ichi Ito^1^, Yuki Sonobe^1^, Kazuyoshi Ikeda^1,2,3^, Kentaro Tomii^2^, Junichi Higo^4,§^

^1^School of Life Sciences, Tokyo University of Pharmacy and Life Sciences, 1432-1 Horinouchi, Hachioji, Tokyo, 192-0392, Japan

^2^Computational Biology Research Center (CBRC), National Institute of Advanced Industrial Science and Technology (AIST), 2-42 Aomi, Koto-ku, Tokyo 135-0064, Japan

^3^PharmaDesign, Inc., 2-19-8 Hacchobori, Chuo-ku, Tokyo 104-0032, Japan

^4^The Center for Advanced Medical Engineering and Informatics, Osaka University, Open Laboratories for Advanced Bioscience and Biotechnology, 6-2-3, Furuedai, Suita, Osaka 565-0874, Japan

# S1 SUPPLEMENTARY METHODS

## S1.1 The method for embedding the inter-cluster network into 3D space

In this subsection, we explain the method for embedding the cluster network into three-dimensional (3D) space according to the adjacency matrix $a_{uv}$ (Eq. 7 in the paper). The accuracy of the obtained 3D distribution is assessed by F-measure as explained in the following subsection. We first define a squared distance between two clusters $u$ and $v$ in the 3D space as:

$d_{uv}=(x_{u}-x_{v})^{2}+(y_{u}-y_{v})^{2}+(z_{u}-z_{v})^{2}$ (S1)

where the 3D positions of the clusters $u$ and $v$ are expressed as $\vec{X}_{u}=[x_{u},y_{u},z_{u}]$ and $\vec{X}_{v}=[x_{v},y_{v},z_{v}]$, respectively. Then, we define an objective function $J$ according to the method by Yamada et al. (2003) as follows:

$J=\sum_{u=1}^{K_{\text{c}}-1} \sum_{v=u+1}^{K_{\text{c}}} E_{uv}+\frac{\mu}{2}\sum_{u=1}^{K_{\text{c}}} (x_{u}^{2}+y_{u}^{2}+z_{u}^{2})$ , (S2)

where $\mu$ is a positive scalar, for which the role is explained below, and $E_{uv}$ is an “interaction energy” between the clusters $u$ and $v$ defined as:

$E_{uv}=\frac{1}{2}a_{uv}d_{uv}-(1-a_{uv})\ln(1-\rho_{uv})$, (S3)

where

$\rho_{uv}=\exp[-\frac{1}{2}d_{uv}]$. (S4)

For a pair of clusters $(u,v)$ with the adjacency matrix elements of $a_{uv}=1$ (Eq. 7 in the paper), the first term of Eq. S3 decreases with decreasing $d_{uv}$ and the second term is always zero. For pairs with $a_{uv}=0$, contrarily, the first term of Eq. S3 is always zero and the second term is repulsive. The second term of Eq. S3 is introduced to confine the cluster distribution in a restricted volume of the 3D space (i.e., the distribution is narrowed with increasing $\mu$).

We randomly distributed the $K_{\text{c}}$ points in the 3D space for the initial positions of clusters, and minimized the objective function with a Newton Raphson method. The converged positions $\text{\{}\vec{X}_{1},\vec{X}_{2},...,\vec{X}_{K_{\text{c}}}\}$ through the minimization provided the 3D distribution (i.e., segment fold universe). We examined different sets of the initial positions, and obtained similar distributions. The distribution reported in Results of the paper is one of them. Last, clusters $u$ and $v$ were linked in the 3D space, when the adjacency matrix $a_{uv}=1$.

## S1.2 F-measure

The linked clusters are closed to one another in the full-dimensional space with satisfying $f(\Delta\bar{C}_{u},\Delta\bar{C}_{v})>0.7$ (i.e., $a_{uv}=1$). Then, we assess whether the linked clusters are close to one another in the 3D space by calculating an F-measure (van Rijsbergen 1979), as follows: First we define a sphere (radius is $r_{3D}$) around the cluster $u$ in the 3D space. Next, precision $P_{u}(r_{3D})$ and recall $R_{u}(r_{3D})$ around the cluster $u$ are defined, as follows:

$P_{u}(r_{3D})=N^{\text{link}}_{u}(r_{3D})/N^{\text{clst}}_{u}(r_{3D})$, (S5)

and

$R_{u}(r_{3D})=N^{\text{link}}_{u}(r_{3D})/N^{\text{link}}_{u}(\infty)$, (S6)

where $N^{\text{clst}}_{u}(r_{3D})$ is the number of clusters involved in the sphere except for the cluster $u$ itself, $N^{\text{link}}_{u}(r_{3D})$ the number of clusters directly linked to the cluster $u$ inside the sphere, and $N^{\text{link}}_{u}(\infty)$ the number of all clusters directly linked to the cluster $u$. The F-measure with respect to the cluster $u$ is given as:

$F_{u}(r_{3D})=\frac{2P_{u}(r_{3D})R_{u}(r_{3D})}{P_{u}(r_{3D})+R_{u}(r_{3D})}$. (S7)

After calculating $F_{u}(r_{3D})$ for each cluster, $F_{u}(r_{3D})$ was averaged over all clusters:

$\bar{F}(r_{3D})=\frac{\sum_{u} F_{u}(r_{3D})}{K_{\text{c}}}$. (S8)

With changing $r_{3D}$, we searched the largest $\bar{F}(r_{3D})$ value, which is designated as $\bar{F}_{\max}$. The larger the $\bar{F}_{\max}$, the better the 3D distribution in reflecting the full-dimensional distribution to the 3D one.

## S1.3 The coloring method for clusters in the 3D network

In the paper, we defined quantities $n_{\alpha}$, $n_{\beta}$, and $n_{\alpha\beta}$ to express the secondary-structure contents for each community. The color of a community in Figure 4, Figure 5, and Figure 6 of the paper is specified by the [R, G, B] color values. The RGB values for a α community are [250, $250-3n_{\alpha}(w)$, $250-3n_{\alpha}(w)$]. Those for a β community are [$250-3n_{\beta}(w)$, $250-3n_{\beta}(w)$, 250]. The indices for a αβ community are [$250-3n_{\alpha}(w)$, 250, $250-3n_{\alpha}(w)$] if $n_{\alpha}(w)>n_{\beta}(w)$ and [$250-3n_{\beta}(w)$, 250, $250-3n_{\beta}(w)$] if $n_{\alpha}(w)<n_{\beta}(w)$. The color for all of the randomly structured communities is black. The color of links connecting clusters within α communities is red, color of those within β communities is blue, and color of those within αβ communities is green. Other links are colored by black.

We used different coloring in Figure 11 in the paper: We applied a single color to the corresponding communities for $K_{\text{c}}=$ 1000, 2000, and 3000. For instance, majority of segments in the orange-colored community of Figure 11A are involved in the orange-colored ones in Figures 11B and 11C.

# S2 SUPPLEMENTARY RESULTS

Figure S1 illustrates a community consisting of fragments that adopt helix-turn-helix structures for $K_{\text{c}}=2000$. The sphere size of a cluster is proportional to the number of constituent segments in the cluster. We exemplify seven clusters, which are numbered from 1 to 7 in Figure S1.


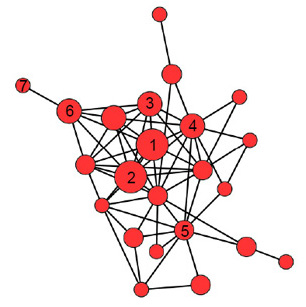


Figure S1 : **A community composed of helix-turn-helix structures**

Figure S2 displays four structures randomly picked from each of the seven clusters, where the N-terminal side of the polypeptide is colored blue. Clusters 1 and 2, which are the central clusters of the community, consist of regular helix-turn-helix. The structures from clusters 3-6 are slightly irregular. The structural irregularity for cluster 7, which is located at a fringe of the community (see Figure S1), is large.


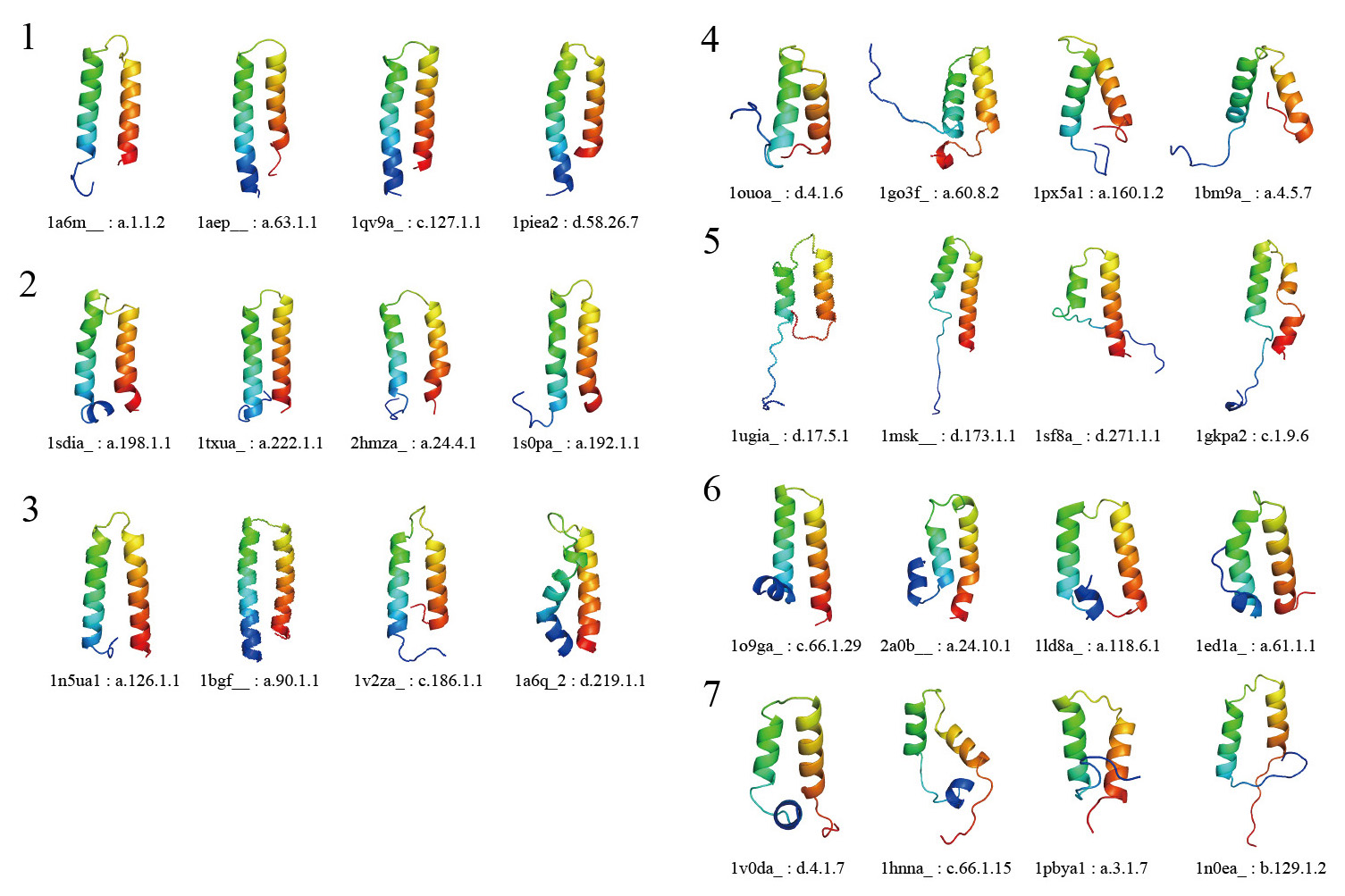

Figure S2 : **Structures belonging to the same cluster**

# S3 SUPPLEMENTARY REFERENCES

Yamada T, Saito K, Ueda N: 2003. In: *Proc Twentieth International Conference Machine Learning* (*ICML*-*2003*). Edited by Fawcett T, Mishra N. Menlo Park: The AAAI Press; 2003:832-839.

van Rijsbergen CJ: *Information retrieval* (*2nd edition*). Newton: Butterworth-Heinemann; 1979.
